# Quantitative X-ray Elemental Imaging in Plant Materials at the Subcellular Level with a Transmission Electron Microscope: Applications and Limitations

**DOI:** 10.3390/ma7043160

**Published:** 2014-04-21

**Authors:** Shaoliang Chen, Heike Diekmann, Dennis Janz, Andrea Polle

**Affiliations:** 1 College of Biological Sciences and Technology, Beijing Forestry University, Beijing 100083, China; E-Mail: lschen@bjfu.edu.cn; 2 Forstbotanik und Baumphysiologie, Büsgen-Institute, Georg-August Universität Göttingen, Göttingen 37077, Germany; E-Mails: heike.diekmann@forst.uni-goettingen.de (H.D.); djanz@gwdg.de (D.J.)

**Keywords:** TEM, EDX, agar standard, *Populus euphratica*, root, cortex, cell wall, vacuole, xylem vessel, salt tolerance

## Abstract

Energy-dispersive X-ray microanalysis (EDX) is a technique for determining the distribution of elements in various materials. Here, we report a protocol for high-spatial-resolution X-ray elemental imaging and quantification in plant tissues at subcellular levels with a scanning transmission electron microscope (STEM). Calibration standards were established by producing agar blocks loaded with increasing KCl or NaCl concentrations. TEM-EDX images showed that the salts were evenly distributed in the agar matrix, but tended to aggregate at high concentrations. The mean intensities of K^+^, Cl^−^, and Na^+^ derived from elemental images were linearly correlated to the concentrations of these elements in the agar, over the entire concentration range tested (*R* > 0.916). We applied this method to plant root tissues. X-ray images were acquired at an actual resolution of 50 nm × 50 nm to 100 nm × 100 nm. We found that cell walls exhibited higher elemental concentrations than vacuoles. Plants exposed to salt stress showed dramatic accumulation of Na^+^ and Cl^−^ in the transport tissues, and reached levels similar to those applied in the external solution (300 mM). The advantage of TEM-EDX mapping was the high-spatial-resolution achieved for imaging elemental distributions in a particular area with simultaneous quantitative analyses of multiple target elements.

## 1. Introduction

Energy-dispersive X-ray microanalysis (EDX) is a technique for analyzing elements at the microscopic level. For this purpose, scanning (SEM) or transmission electron microscopes (TEM) are equipped with an energy dispersive system for quantitative electron probe X-ray microanalysis. The TEM-EDX system requires embedded samples, which enable high spatial resolution. The SEM-EDX system can be applied to surfaces of untreated specimens and, thus, provides a rapid way of measuring elemental distributions in plant and animal materials. The most significant recent advance has been the development of cryo-SEM for *in situ* elemental quantification by EDX [1,2]. This method assumes that little or no element redistribution can occur in a frozen-hydrated specimen [1,2]. Thus, SEM-EDX allows direct analysis of frozen-hydrated materials without freeze-drying or embedding. However, in general, the low spatial resolution of SEM-EDX makes it difficult to determine structures from the rough surfaces of frozen-hydrated bulk specimens. The cellular and subcellular distributions of elements in biological materials are typically investigated at relatively high spatial resolution with TEM-EDX [3–5]. This technique requires preparation of thin sections of resin-embedded plant materials [6–8]. For element analyses, TEM-EDX protocols have been developed that avoid ion re-distribution during the embedding procedure [6]. Thus, TEM-EDX can be used to examine elements of interest within cell compartments with high spatial resolution.

The advent of imaging techniques has advanced the analysis of elemental distributions and the quantification of elements in cells and tissues [2]. Elemental imaging also provides improved spatial information for the analysis of biological materials. Moreover, the mean values of elemental concentrations derived from an X-ray image represent several hundreds to thousands of probe measurements, which provide more reliable information than dozens of randomly selected measurement points. Previous studies have described results from frozen-hydrated biological materials analyzed with X-ray imaging in a SEM system [9,10]. However, quantitative elemental imaging in a TEM system has seldom been attempted in biological materials.

For quantification of X-ray images, it is necessary to obtain standards that contain the element of interest in known amounts. It was previously established that calibration standards for quantitative X-ray microanalysis in a TEM could be produced by adding 6–600 mM KCl to 5% agar [7]. The agar-KCl blocks proved to be highly suitable for the quantification of X-ray microanalytical measurements [7]. In the present study, we prepared calibration standards by adding 0–320 mM KCl or NaCl to an agar matrix. The samples were processed in the same way as the plant tissues; *i.e.*, they were rapidly frozen, freeze-dried, embedded in plastic, and sectioned at 1.0-μm thickness [6–8]. Elemental images of agar standards were quantified and the data were used to generate calibration curves for assessing element concentrations in plant cell compartments.

EDX analyses are particularly suited to investigations of stresses imposed by toxic elements or excess salinity [2]. Here, we employed TEM-EDX to study salt distributions in *Populus euphratica* Oliver, a salt-tolerant woody species. *P. euphratica* is used as a model plant to address tree-specific mechanisms underlying salt tolerance [11–20]. Previous X-ray microanalysis with random point measurements revealed that, compared to salt-sensitive species, *P. euphratica* roots accumulated more Na^+^ in cortical cell walls, but significantly less Na^+^ in stelar walls [21,22]. Furthermore, vacuolar compartmentalization of Na^+^ and Cl^−^ could be demonstrated in root cortical cells [22], but the concentrations were apparently lower in vacuoles than in the cell walls [21,22]. However, those results were somewhat difficult to interpret, because the images compared were not acquired under the same measuring conditions. In these studies, to obtain representative data for the analyzed probe, the electron beam was adjusted to the size of the investigated structure [21,22]. Thus, probe measurements of cell walls, cytoplasm, and xylem vessels were acquired with a narrow electron beam, and measurements of vacuoles were acquired with a broad electron beam that covered the vacuolar lumen [21–25]. Therefore, the data had to be corrected for the different measuring intensities of the applied electron beam.

In the present study, an electron beam of uniform width and intensity was used for quantitative X-ray elemental imaging of root cells of *P. euphratica*. With the use of standards, we estimated the ion concentrations within biological materials, including the root cortical cells and xylem vessels. The use of cryo-EDX to investigate frozen hydrated samples directly with SEM avoids potential drying artifacts that may occur during freeze drying. However, it precludes structure determinations in the elemental images, due to its relatively low resolution. In our study, agar standard and plant samples were analyzed at a high-spatial resolution ranging from 50 nm × 50 nm to 100 nm × 100 nm. High-spatial resolution elemental images of these tissues showed that the ion gradient varied between different subcellular compartments. High-spatial resolution of intracellular ion concentrations is a major advantage of TEM-EDX compared to the lower resolution elemental images obtained with cryo-analytical SEM. Therefore, the proposed TEM-EDX protocol is a feasible method for estimating multiple elemental concentrations within cell compartments.

## 2. Results and Discussion

### 2.1. Elemental Images of Agar Standards

**Figure 1.**
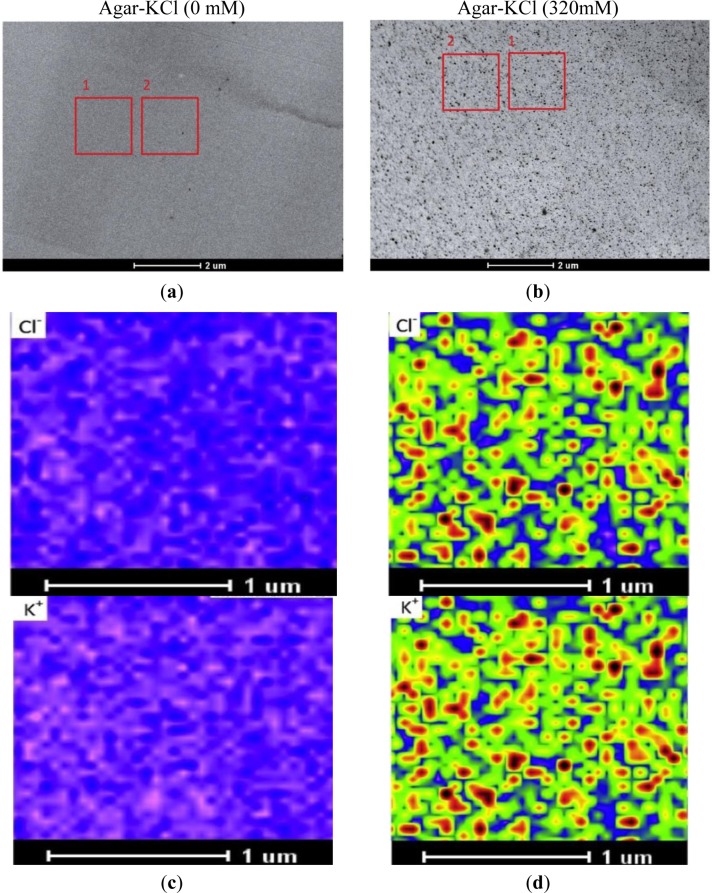
Representative STEM images (**a**,**b**) and elemental images (**c**,**d**) of 5% agar containing 0 or 320 mM KCl. STEM images with frame 1 showing the area for drift correction and frame 2 the measuring area. Elemental images of X-ray intensities with the scale fixed between the maximum (black) and minimum pixel intensities (violet) in a range of 25–1000 for Cl^−^ and 20–1000 for K^+^.

STEM images of specimens were acquired prior to X-ray imaging. In the STEM image, a frame adjacent to the measured region was used for drift correction (Figure 1A,B); this is required, because micrographs at high magnification are distorted by motion of the sample during the scanning and image acquisition. For the purpose of this study, agar specimens were analyzed in an area of 1.75 μm × 1.75 μm, at a spatial resolution of 50 nm × 50 nm (35 × 35 data points), with a dwell time of 10 s per point. X-rays were detected with the EDX detector at 80 kV with a low current (beam size set to 8 in the Tecnai TEM, FEI, Hillsboro, OR, USA). The beam current was constant during the collection of elemental maps, and each collection period lasted at least 4 h. Drift corrections were performed automatically every 400 live seconds during the period of X-ray imaging. Kα line peak area intensities (counts) of the measuring regions were analyzed with TEM imaging and analysis (TIA) software (FEI, Hillsboro, OR, USA). The obtained elemental images were evaluated with a rainbow color scale, where X-ray intensity increases from pink (low intensity) to blue, green, yellow, red, and black (high intensity). To visualize changes at low and high concentration ranges, we used log-scaling. The maximum, mean, and minimum intensities of K^+^ and Cl^−^ from the measured regions were extracted with TIA software. The maximum and minimum values must be fixed for comparing concentrations between different elemental images. In this study, the maximum value was set at 1000, due to the high pixel intensity measured at 320 mM KCl agar. To set the minimum limit, we used the mean value of minimum intensities across the elemental images.

In the absence of KCl, STEM images of agar blocks showed a uniform, resin-embedded agar matrix (Figure 1A). In the presence of KCl, electron-dense precipitates became visible in the agar block (Figure 1B). X-ray imaging indicated that these aggregates were formed from KCl (Figure 1C,D). Thus, the sublimation of water apparently resulted in KCl aggregation and the formation of crystals. During sample preparation, we took rigorous precautions to minimize ion migration. We used diethyl ether, the preferred substitution solvent, in the vacuum infiltration process. Prior to the infiltration step, a molecular sieve was used to absorb any water in the diethyl ether. Moreover, the relative humidity was maintained at about 10%–20% to exclude atmospheric moisture during the embedding and sectioning processes. All sectioned specimens were coated with carbon under vacuum, and subsequently, stored in a dry box until analysis. Our results showed that the high intensity points were evenly distributed in the agar matrix (Figure 1B); thus, large-scale migration of KCl did not occur during the freezing, freeze-drying, and embedding processes. Notably, we observed perfect overlap of the K^+^ and Cl^−^ images (Figure 1D).

**Figure 2.**
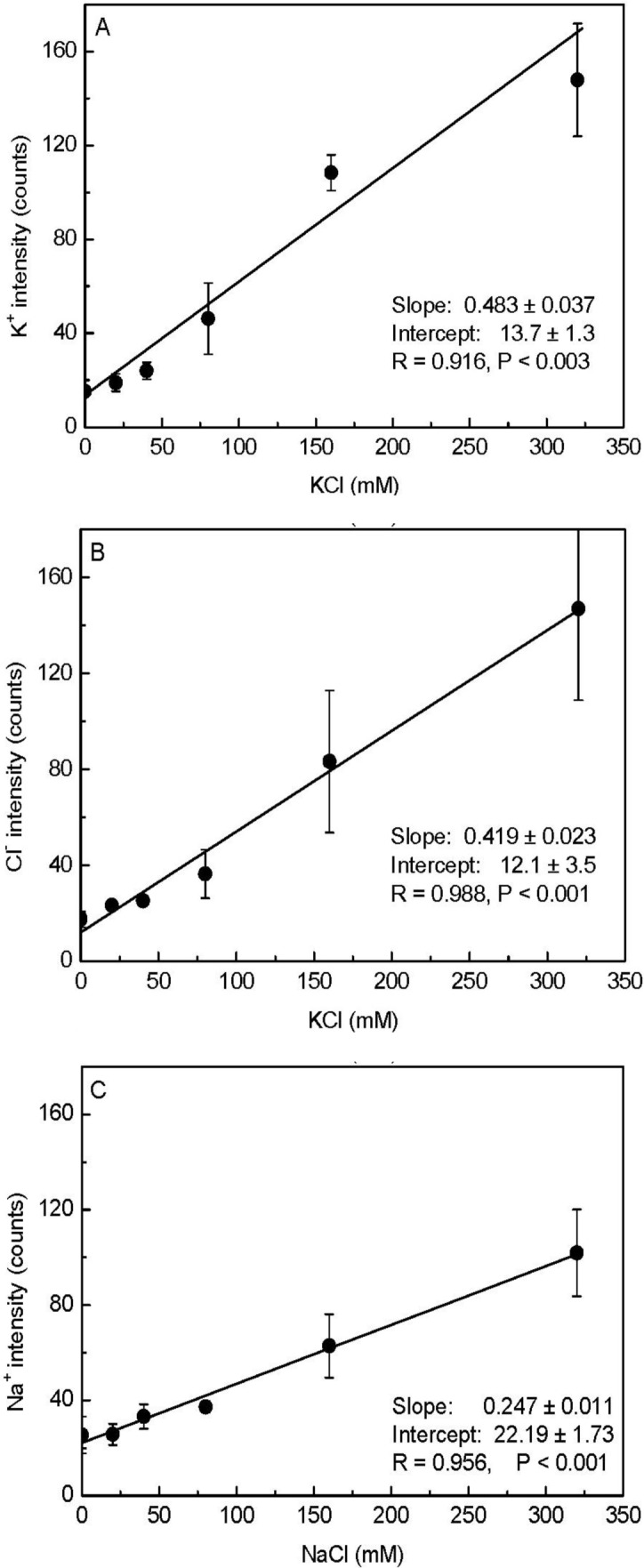
Correlation between pixel intensities calculated from elemental images and the amounts of K^+^ (**A**), Cl^−^ (**B**), and Na^+^ (**C**) present in the agar blocks. Data were fitted by linear regression. Data represent means ± SD (*n* = 4).

As shown in Figure 1D, the pixel intensities of potassium and chloride varied throughout the X-ray images. Therefore, taking the minimum intensity as the background, we calculated the mean values corrected for the minimum intensity (*i.e*., element peak = mean intensity − minimum intensity) for each elemental image (35 × 35 data points) to establish the calibration curve. When the measured K^+^ and Cl^−^ intensities were plotted against the added KCl concentrations, a linear correlation was observed between the mean intensity and the content of these elements in the agar matrix (Figure 2). We noticed that the fitted regression lines for K^+^ and Cl^−^ failed to go through the origin (Figure 2). This would be expected for agar that contained insoluble K^+^ and Cl^−^ prior to the addition of KCl. However, in agar blocks without KCl, we did not find evident peaks of either K^+^ or Cl^−^ in the measured spectra (Figure 1C). Therefore, the intercept represents background noise.

We also prepared a NaCl-agar standard series with the same protocol as above. When the measured Na^+^ intensities were plotted against added NaCl (0 to 320 mM), the slope of the Na^+^ regression line (0.25) was nearly half those derived for K^+^ (0.48) and Cl^−^ (0.42; Figure 2); this indicated that our assay had less sensitivity for Na^+^. As a result, there was higher uncertainty in the background signal for Na^+^ concentrations compared to K^+^ concentrations. Based on the error observed in the Y-intercepts, the detection limits for the elements were 3 mM K^+^, 8 mM Cl^−^, and 7 mM Na^+^. Note that these limits show that the method had high sensitivity, because concentrations of about 10 mM correspond to only 0.01 fmol·μL^−1^ when converted to the volumes analyzed here. The increase in the standard deviation (SD) with increasing salt concentrations was caused by aggregation of KCl or NaCl (Figure 1D, Figure 2) resulting in large differences between the pixel intensities detected from the areas in the presence and absence of crystals.

**Figure 3.**
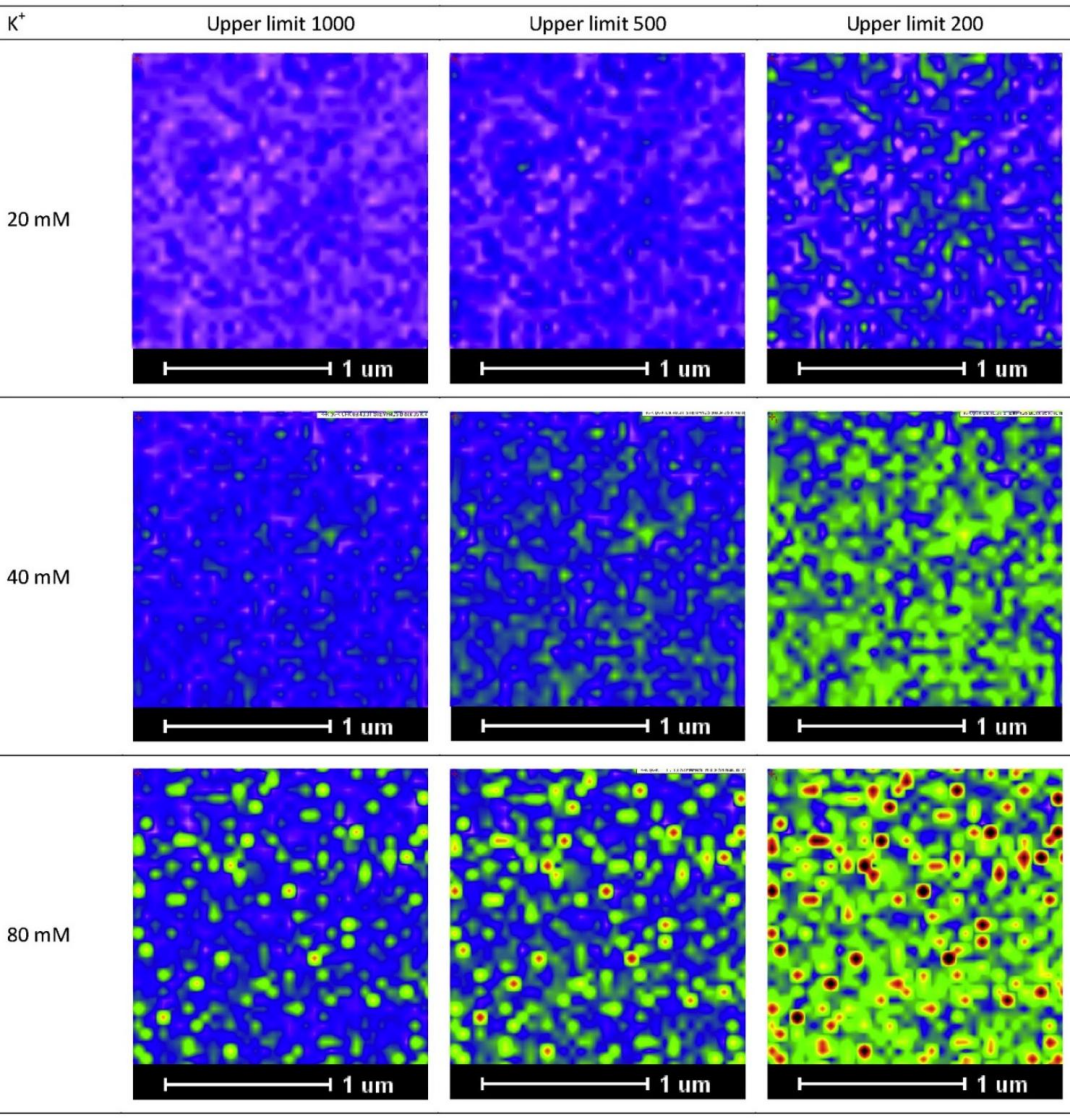
Representative K^+^ images of 5% agar containing 20, 40, and 80 mM KCl. The distributions of elements at low concentrations were enhanced by adjusting the scaling. Images are shown for maximum values (upper limits) of 1000, 500, or 200. In all cases, the minimum value was fixed at 20.

As a practical note, we would like to add that visualization can be enhanced by adjusting the intensity scale for the image at different concentrations. For example, for image profiles of low K^+^ concentrations, visualization of the K^+^ distribution can be enhanced by reducing the maximum intensity from 1000 to lower values, as demonstrated in Figure 3.

### 2.2. Elemental Images of P. euphratica Root Cells

**Figure 4.**
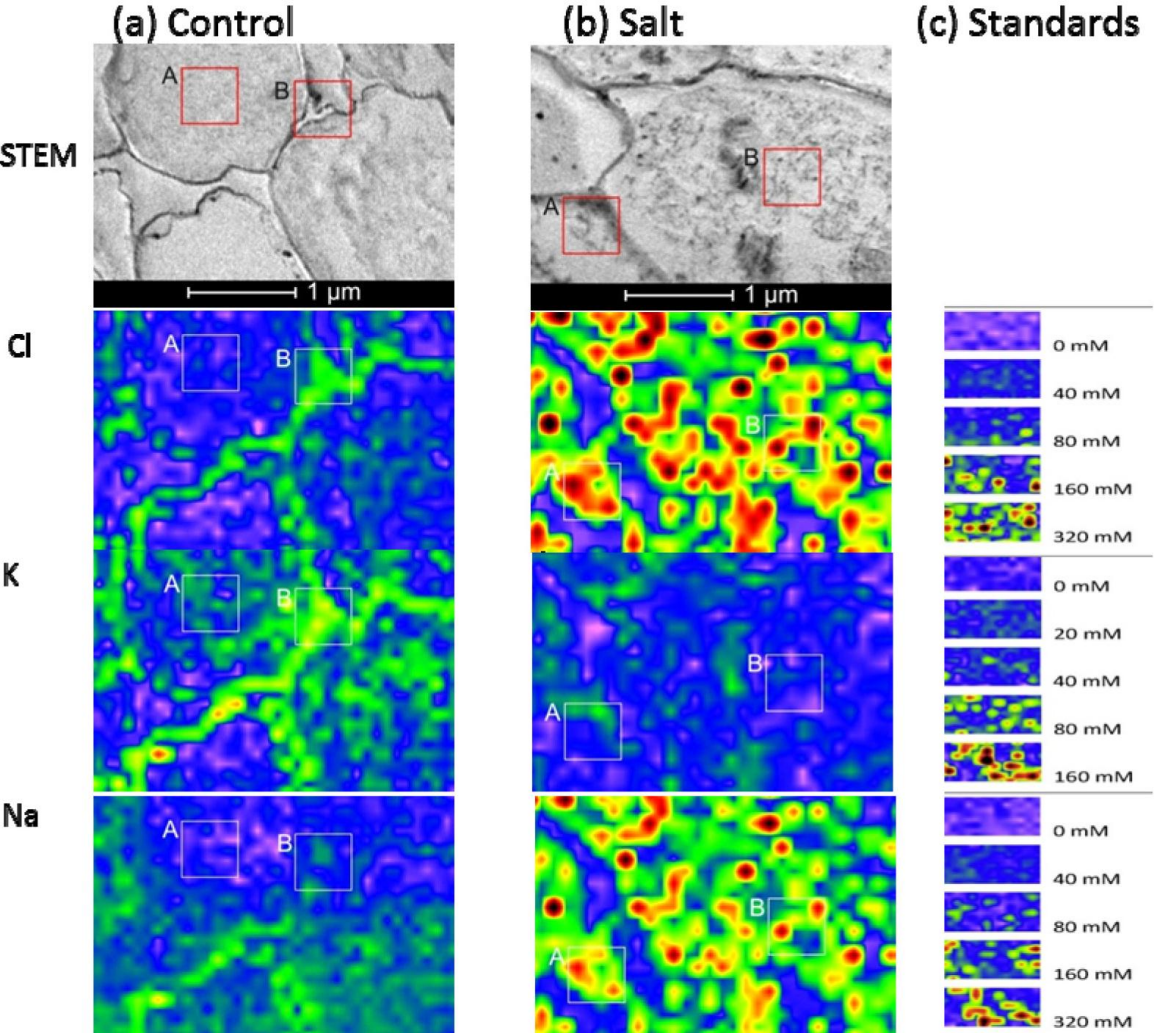
Representative STEM images and elemental images of root cortex in control (**a**) and NaCl-treated *P. euphratica* (**b**). For X-ray imaging, the area measured in root cortical cells depended on the cell structures of interest. The spatial resolution ranged from 50 nm × 50 nm to 100 nm × 100 nm. Elemental images of 5% agar standards (**c**) were acquired at the same resolutions. The maxima were set to 1000 for Na^+^ and Cl^−^ and 500 for K^+^; the minima were set to 30 for Na^+^, 25 for Cl^−^, and 10 for K^+^. Frames A and B were used to calculate the mean intensities (Table 1).

**Figure 5.**
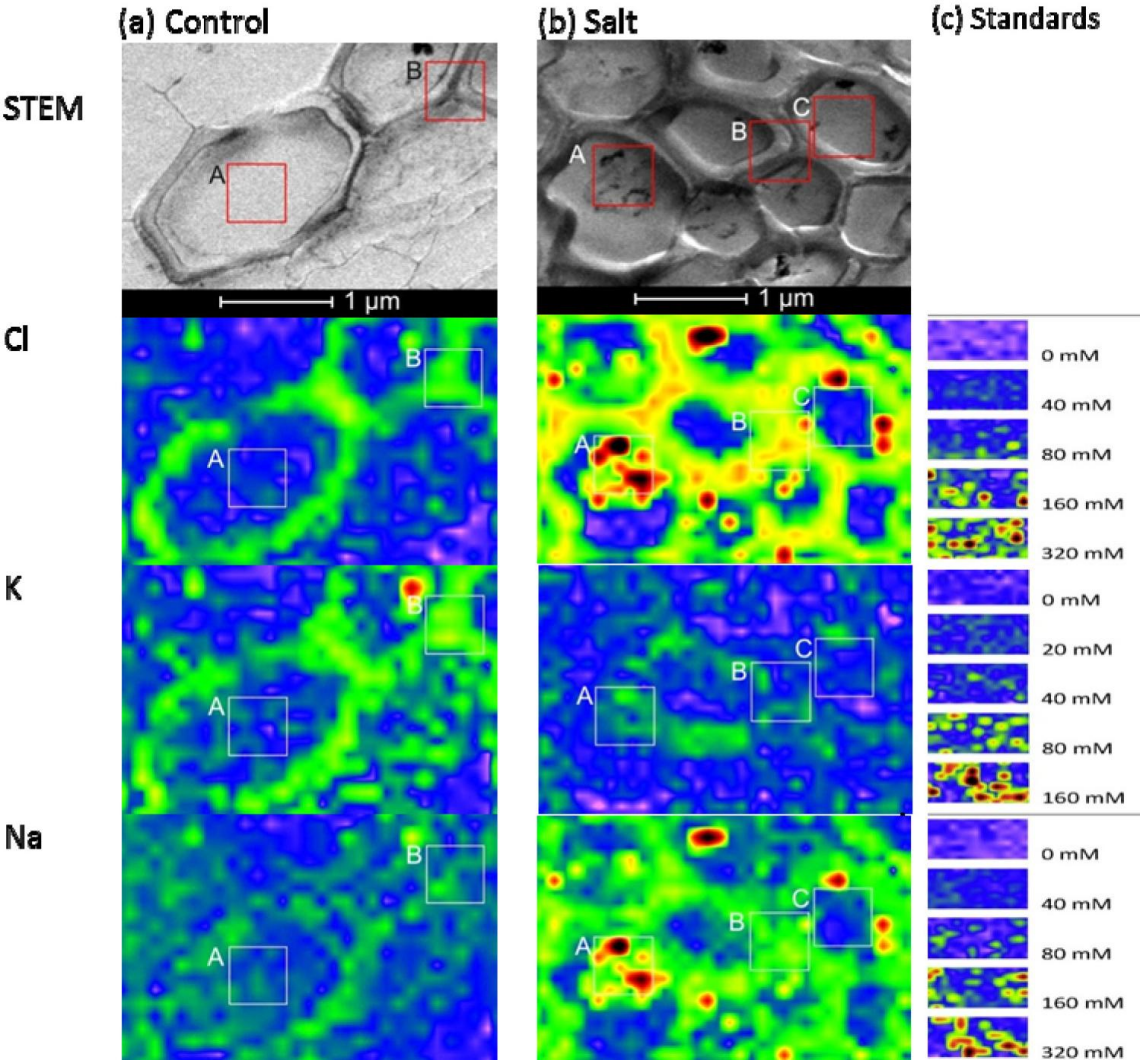
Representative STEM images and elemental images of xylem vessels in control (**a**) and NaCl-treated *P. euphratica* (**b**). For X-ray imaging, the areas measured in xylem vessels depended on the cell structures of interest. The spatial resolution ranged from 50 nm × 50 nm to 100 nm × 100 nm. Elemental images of 5% agar standards (**c**) were acquired at the same resolutions. The maximum and minimum pixel intensities were fixed in these images for agar and root specimen as described in Figure 4. Frames A, B, and C were used to calculate the mean intensities (Table 1).

Tissue and cellular studies have repeatedly shown that *P. euphratica* is able to control K^+^/Na^+^ homeostasis under salt stress [26–29]. In this study, *P. euphratica* plants were cultured in hydroponic Long Ashton nutrient solution. After salt shock with 300 mM NaCl for 24 h, we analyzed K^+^, Cl^−^, and Na^+^ distributions in the root cortex and xylem vessels with X-ray elemental imaging in a TEM, and compared the results with those of untreated controls (Figures 4 and 5). The area measured depended on the cell structures of interest. For X-ray imaging at the cellular level, the actual spatial resolution ranged from 50 nm × 50 nm to 100 nm × 100 nm. Elemental images of agar standards were obtained at the same resolution as that used for the plants (Figures 4 and 5). The measuring time was 10 s for each point.

When plants were grown under control conditions, high-spatial resolution elemental images of root cortex cells showed that both Cl^−^ and K^+^ were higher in the cortical wall than in the vacuole. These semi-quantitative assessments of the elemental concentrations were based on the scales produced by the agar standards (Figure 4). For quantitative estimations, intensities were measured in the areas indicated with the frames in Figure 4, and the data are compiled in Table 1. Based on these data and the calibration curves (Figure 2) we estimated that the cell wall contained 67 mM Cl^−^ and 50 mM K^+^ and the vacuoles contained 14 mM Cl^−^ and 13 mM K^+^ in cortical cells of controls. In contrast, the Na^+^ concentrations in the vacuoles of root cells were indistinguishable from background noise (3.6 mM), and the cell walls contained low Na^+^ concentrations (19 mM) (frame B, Figure 4a). Given the small volumes measured, it was difficult to quantify low element concentrations precisely, because the signal was not sufficiently distinguished from the background.

When plants were exposed to salt shock, they accumulated high levels of both Na^+^ and Cl^−^ in cell walls and in the vacuoles of root cortex cells (Figure 4b). The concentrations exceeded those used to produce the calibration curves, but we estimated that they would correspond roughly to 386 mM Cl^−^ and 517 mM Na^+^ in the vacuole and 429 mM Cl^−^ and 586 mM Na^+^ in the cell wall, assuming a linear relationship between the elemental concentrations and the intensities measured in the high concentration range (Figure 4b, Table 1). The concordance of Na^+^ and Cl^−^ observed at this resolution indicated that salt aggregates had formed in the vacuole (Figure 4b), due to the non-aqueous preparation technique. Aggregate formation may result in overestimations of salt concentrations at small scales.

In contrast to Na^+^ and Cl^−^, salt shock lowered the K^+^ levels in root cortex cells. However, note that the measurements acquired in the frames shown in Figure 4 (Frame A: 36 mM, Frame B: 24 mM) indicated K^+^ concentrations were well above the background level.

We also imaged the vessels in the vascular system of *P. euphratica* roots at high spatial resolution (Figure 5). The lumina of the vessels of unstressed plants contained K^+^ and Cl^−^ concentrations at the detection limit, but the estimated Na^+^ concentration (27 mM) was well above the detection threshold (Figure 5a). In the vessel walls, the analyzed elements were enriched (30–40 mM). After salt shock, the concentrations of Na^+^ and Cl^−^ in the vessel lumina increased dramatically (197 mM Na^+^ and 98 mM Cl^−^), and the concentrations of K^+^ slightly increased (39 mM, Figure 5b). Vessel walls of salt shocked plants contained about three times higher Na^+^ and Cl^−^ concentrations than the lumen (Figure 5b). In the lumina, we also detected salt aggregates, most likely caused by the sublimation of water during non-aqueous sample preparation (Figure 5b, Frame A). These aggregates contained estimated concentrations of about 750 mM Cl^−^ and 949 mM Na^+^. Apparently, at high concentrations salt tended to aggregate; this phenomenon may have led to overestimating salt concentrations in the biological systems, when the analysis was conducted at very high resolution. It is necessary to analyze the whole region of target structure, which includes not only the salt aggregates but also the areas without aggregates. In our analysis, the aggregates filled about half of the lumen of the vessel; thus, we may assume that the true salt concentration in the xylem sap was correspondingly, *i.e.*, twice lower than the measured value.

The capacity to maintain K^+^ homeostasis is crucial for herbaceous and woody species to adapt to saline environments [20,30]. We found that the loss of K^+^ induced by salt shock in *P. euphratica* plants was not pronounced in the measured cell compartments compared to the increment of salt ions (Figures 4 and 5). This was consistent with our previous findings that *P. euphratica* plants exhibit a remarkable ability to retain K^+^ under saline conditions [11], presumably due to their relatively high rates of net uptake and transport of K^+^ [22]. The observed enrichments of Na^+^ and Cl^−^ were previously reported for *P. euphratica* roots exposed to high salinity [21,22]. The vacuolar compartmentalization of salts (as detected here in cortical cells) is crucial for *P. euphratica* to adapt to saline environments [20]. The observed high concentrations in both the cell walls and the vacuoles suggest that exposure to salt shock initially flushed the roots with salt. This notion was supported by the observation of very high salt concentrations in the vessel lumina. In our previous microanalysis, we found that *P. euphratica* accumulated higher Na^+^ and Cl^−^ in the cortical walls than in the vacuole, and that the salt concentration declined in the vascular system [21,22]. It is likely that the suberized endodermis of *P. euphratica* blocked apoplastic ion transport into the inner root, and vacuole sequestration may also have restricted symplastic translocation of ions from the cortex to the xylem [21,22]. However, in those studies, the exposure conditions differed, because the *P. euphratica* were grown in soil [21–23]. Moreover, a time course analysis of *P. euphratica* under salt stress showed Na^+^ accumulation and redistribution over the long term [14]. *P. euphratica* roots exhibit a high capacity for Na^+^ extrusion across the plasma membrane under NaCl stress, which results in a Na^+^ gradient between the apoplast and the symplast [26,27]. However, the present results showed that non-acclimated *P. euphratica* roots could not avoid symplastic salt accumulation, and high transport into the xylem occurred with exposure to excessive salinity.

Recently, specific fluorescent probes have been employed to trace the distribution of elements such as Ca^2+^, Na^+^ and others. While these probes are well suited to investigate cell cultures or other thin-walled cell types [17,28,29], they cannot penetrate tissues with thick cell walls. An advantage of the present method is its applicability to these tissues. Moreover, we can measure simultaneously the localization of multiple elements.

## 3. Experimental Section

### 3.1. Plant Materials and Treatment

Hydroponically cultured plants of *P. euphratica* (clone B2) were used in this study. The stock culture, obtained from *P. euphratica* trees grown in the Ein Avdat Valley (Israel), was multiplied by micropropagation [31]. Rooted plantlets were transferred to aerated hydrocultures supplemented with Long Ashton nutrient solution for 3 months [14]. The plants were maintained in a growth room at 24 °C. A 16 h photoperiod was maintained with cool-white fluorescent light, which provided about 200 μmol quanta m^−2^·s^−1^ of photosynthetically active radiation. Uniform plants of 80–100 cm in height with 40–60 leaves were subjected to salt shock with 300 mM NaCl for 24 h. Control plants were cultured in Long Ashton nutrient solution without addition of NaCl.

### 3.2. Root Sample Preparation

Our standard procedure for sample preparation followed the protocol of Fritz [6]. Briefly, root apices (*ca.* 1.5 cm long) were sampled from control and salt stressed *P. euphratica* plants. The root samples were immediately placed in aluminium sample holders and rapidly frozen in a 2:1 mixture of propane:isopentane, which was cooled with liquid nitrogen. Samples were vacuum freeze-dried at −60 °C for 120 h, and then, slowly equilibrated to the room temperature (22 °C) over a period of 24 h. Then, the samples were stored over silica gel until plastic infiltration and polymerization.

Before plastic infiltration, freeze-dried root segments were transferred into vacuum-pressure chambers, and infiltrated with ether at 27 °C overnight. The plastic preparation was a 1:1 mixture of styrene and butyl methacrylate, with 1% benzoylperoxide, stabilized with 50% phthalate. Plastic infiltration was conducted in three steps: samples were incubated in a 1:1 mixture of ether and plastic for 4 h; then, they were transferred to a 1:3 mixture of ether and plastic for 4 h; finally, they were transferred to 100% plastic for 24 h (×2). Following infiltration, samples were transferred into gelatin capsules, polymerized at 60 °C for 12 h, then transferred into an oven and polymerized at 30 °C for at least 7 days.

### 3.3. Preparation of Agar Standards

In this study, we used purified agar as the supporting matrix for standardized element concentrations. Agar is an organic material and may ideally undergo shrinkage similar to that observed in biological tissues during freezing and freeze-drying [7,32]. KCl and NaCl agar standards were established as previously described [7,25]. Briefly, 10 g agar (Sigma, Steinheim, Germany) was suspended in 2 L of de-ionized water at room temperature. To remove ionic impurities, the water was replaced once a day with freshly de-ionized water. After 10 days of purification, the agar was freeze-dried at −60 °C for 96 h and stored at room temperature. The required amounts of salts for the KCl and NaCl series (0, 20, 40, 80, 160, 320 mM) were dissolved in heated water with 5% purified agar, w/v. Then, the solutions of agar with different salt concentrations were poured in 2 mm-thick layers in Petri dishes. Blocks of 1–2 mm in length and width were cut with a razor blade, immediately placed into baskets made of fine aluminum mesh, and rapidly frozen in a mixture of propane:isopentane (2:1) at the temperature of liquid nitrogen. Samples were vacuum freeze-dried at −60 °C for 72 h, and then, they were slowly allowed to equilibrate to room temperature (*ca*. 22 °C) over a period of 24 h. Agar blocks were vacuum-pressure infiltrated with water-free diethyl ether, and then, infiltrated with plastic as described above.

### 3.4. Cutting Specimen Sections

After polymerization, root and agar samples were cut into 1.0-μm-thick sections with a dry glass knife on an ultramicrotome (Ultracut E, Reichert-Jung, Vienna, Austria). Root cross sections were cut in the region approximately 1.0–1.5 cm behind the root apex, which contained few primary xylem vessels. Slices were mounted onto copper grids (mesh 100), coated with carbon, and stored over silica gel until analysis.

### 3.5. X-ray Imaging in a TEM

Root and agar sections were analyzed in a Tecnai™ TEM (Tecnai G^2^ Spirit, FEI Company, Hillsboro, OR, USA) equipped with an EDX detector (EDAX International, Mahwah, NJ, USA). Prior to the X-ray imaging, samples were exposed to high beam current to stabilize the sections. However, the exposure was usually less than 5 min which could not cause severe damages. Quantitative elemental images of agar and root specimens were analyzed with TIA software version 3.2 (FEI Company, Hillsboro, OR, USA). TIA Smart Phase imaging provides improved analyses by automatically collecting spectra and generating phase maps with elemental distributions and associated spectra. STEM images of agar and root specimens were acquired prior to X-ray imaging. In the STEM images, root cells with clear cortical structure and xylem vessels were selected for X-ray imaging. Elemental images of the agar and root specimens were obtained at 1200-fold magnification. X-rays were detected with the EDX detector at 80 kV with a low current (beam size was set to 8 in the Tecnai TEM). The operating parameters were as follows: accelerating voltage: 80 keV; take-off angle: 15°, and the time for collecting X-rays was 10 s for each measuring point. Agar samples containing various salt concentrations were analyzed over an area of 1.75 μm × 1.75 μm at a resolution of 35 × 35 pixels. The maximum, mean, and minimum intensity of K^+^, Na^+^, and Cl^−^ were measured in the indicated regions (frames) and calculated with TIA imaging and analysis software. For area imaging of root tissues, the actual spatial resolution ranged from 50 nm × 50 nm to 100 nm × 100 nm. Elemental images of agar standards were obtained at the same resolutions. During the period of X-ray imaging, drift corrections were performed automatically every 400 s. The minimum intensity was taken as the background, and the mean intensity was extracted by TEM Imaging & Analysis from the measured regions. We used the following formula to calculate the element peak: element peak = the mean intensity − minimum intensity.

## 4. Conclusions

With the use of suitable agar standards, we assessed the concentrations and distributions of diffusible elements in biological materials. The TEM-EDX imaging technique was successfully applied to microtome-sectioned samples of uniform thickness. The advantage of X-ray imaging in a TEM is the high-spatial resolution imaging of multiple elements within an area of interest and its applicability to tissues which are unsuitable for the use of fluorescent probes. The elemental images of agar standards provided a feasible means to make simultaneously semi-quantitative estimations of the concentrations of multiple elements in different cell compartments. The extracted mean values from selected regions of elemental images could be used for quantification, because the pixel intensities of K^+^, Na^+^, and Cl^−^ were linearly correlated with the concentrations of these elements in the agar matrix.

## Figures and Tables

**Table 1.** Mean element concentrations (mM) measured in root cells of *Populus euphratica* grown under control conditions or exposed to salt shock. The mean concentrations are shown for measured areas in cortex cell walls and vacuoles of Figure 4, and in the vascular system cell walls and vessel lumina of Figure 5. Frames refer to those shown in the indicated figures. Salt shock was imposed by adding 300 mM NaCl to the hydroponic nutrient solutions.

**Element**	**Figure 4a: Control**		**Figure 4b: Salt**	

	Vacuole (Frame A)	Cell Wall (Frame B)	Vacuole (Frame B)	Cell Wall (Frame A)
Cl	14	67	386	429
K	13	50	24	36
Na	3	19	517	586

Element	Figure 5a: Control		Figure 5b: Salt		

	Lumen (Frame A)	Cell Wall (Frame B)	Aggregates in lumen (Frame A)	Lumen (Frame C)	Cell Wall (Frame B)
Cl	7	42	750	98	364
K	1	27	58	39	50
Na	27	44	949	197	375

## References

[1] Ryan M.H., McCully M.E., Huang C.X. (2007). Relative amounts of soluble and insoluble forms of phosphorus and other elements in intraradical hyphae and arbuscules of arbuscular mycorrhizas. Funct. Plant Biol.

[2] McCully M.E., Canny M.J., Huang C.X., Miller C., Brink F. (2010). Cryo-scanning electron microscopy (CSEM) in the advancement of functional plant biology: energy dispersive X-ray microanalysis (CEDX) applications. Funct. Plant Biol.

[3] Sauberman A.J., Heyman R.V. (1987). Quantitative digital X-ray imaging using frozen hydrated and frozen dried tissue sections. J. Microsc.

[4] LeFurgey A., Davilla S.D., Kopf D.A., Sommer J.R., Ingram P. (1992). Real-time quantitative elemental analysis and mapping: Microchemical imaging in cell physiology. J. Microsc.

[5] Bidwell S.D., Crawford S.A., Woodrow I.E., Sommer-Knudsen J., Marshall A.T. (2004). Sub-cellular localization of Ni in the hyperaccumulator, *Hybanthus floribundus* (Lindley) F. Muell. Plant Cell Environ.

[6] Fritz E. (1989). X-ray microanalysis of diffusible elements in plant cells after freeze-drying, pressure-infiltration with ether and embedding in plastic. Scanning Microsc.

[7] Fritz E., Jentschke G. (1994). Agar standards for quantitative X-ray microanalysis of resin-embedded plant tissues. J. Microsc.

[8] Fritz E. (2007). Measurement of cation exchange capacity (CEC) of plant cell walls by X-ray microanalysis (EDX) in the transmission electron microscope. Microsc. Microanal.

[9] Marshall A.T., Xu W. (1998). Quantitative elemental X-ray imaging of frozen-hydrated biological samples. J. Microsc.

[10] Marshall A.T., Goodyear M.J., Crewther S.G. (2012). Sequential quantitative X-ray elemental imaging of frozen-hydrated and freeze-dried biological bulk samples in the SEM. J. Microsc.

[11] Chen S., Li J., Wang S., Hüttermann A., Altman A. (2001). Salt, nutrient uptake and transport, and ABA of *Populus euphratica*: A hybrid in response to increasing soil NaCl. Trees Struct. Funct.

[12] Chen S., Li J., Wang T., Wang S., Polle A., Hüttermann A. (2002). Osmotic stress and ion-specific effects on xylem abscisic acid and the relevance to salinity tolerance in poplar. J. Plant Growth Regul.

[13] Gu R., Fonseca S., Puskás L.G., Hackler L.J., Zvara A., Dudits D., Pais M.S. (2004). Transcript identification and profiling during salt stress and recovery of *Populus euphratica*. Tree Physiol.

[14] Ottow E.A., Brinker M., Teichmann T., Fritz E., Kaiser W., Brosché M., Kangasjärvi J., Jiang X., Polle A. (2005). *Populus euphratica* displays apoplastic sodium accumulation, osmotic adjustment by decreases in calcium and soluble carbohydrates, and develops leaf succulence under salt stress. Plant Physiol.

[15] Wang R., Chen S., Deng L., Fritz E., Hüttermann A., Polle A. (2007). Leaf photosynthesis, fluorescence response to salinity and the relevance to chloroplast salt compartmentation and anti-oxidative stress in two poplars. Trees Struct. Funct.

[16] Wang R., Chen S., Zhou X., Shen X., Deng L., Zhu H., Shao J., Shi Y., Dai S., Fritz E. (2008). Ionic homeostasis and reactive oxygen species control in leaves and xylem sap of two poplars subjected to NaCl stress. Tree Physiol.

[17] Sun J., Zhang X., Deng S., Zhang C., Wang M., Ding M., Zhao R., Shen X., Zhou X., Lu C. (2012). Extracellular ATP signaling is mediated by H_2_O_2_ and cytosolic Ca^2+^ in the salt response of *Populus euphratica* cells. PLoS ONE.

[18] Ding M., Hou P., Shen X., Wang M., Deng S., Sun J., Xiao F., Wang R., Zhou X., Lu C. (2010). Salt-induced expression of genes related to Na^+^/K^+^ and ROS homeostasis in leaves of salt-resistant and salt-sensitive poplar species. Plant Mol. Biol.

[19] Janz D., Behnke K., Schnitzler J.P., Kanawati B., Schmitt-Kopplin P., Polle A. (2010). Pathway analysis of the transcriptome and metabolome of salt sensitive and tolerant poplar species reveals evolutionary adaption of stress tolerance mechanisms. BMC Plant Biol.

[20] Chen S., Polle A. (2010). Salinity tolerance of *Populus*. Plant Biol.

[21] Chen S., Li J., Fritz E., Wang S., Hüttermann A. (2002). Sodium and chloride distribution in roots and transport in three poplar genotypes under increasing NaCl stress. Forest Ecol. Manage.

[22] Chen S., Li J., Wang S., Fritz E., Hüttermann A., Altman A. (2003). Effects of NaCl on shoot growth, transpiration, ion compartmentation and transport in regenerated plants of *Populus euphratica* and *Populus tomentosa*. Can. J. Forest Res.

[23] Chen S., Zommorodi M., Fritz E., Wang S., Hüttermann A. (2004). Hydrogel modified uptake of salt ions and calcium in *Populus euphratica* under saline conditions. Trees Struct. Funct.

[24] Ma X., Deng L., Li J., Zhou X., Li N., Zhang D., Lu Y., Wang R., Sun J., Lu C. (2010). Effect of NaCl on leaf H^+^-ATPase and the relevance to salt tolerance in two contrasting poplar species. Trees Struct. Funct.

[25] Chen S., Olbrich A., Langenfeld-Heyser R., Fritz E., Polle A. (2009). Quantitative X-ray microanalysis of hydrogen peroxide within plant cells. Microsc. Res. Tech.

[26] Sun J., Chen S., Dai S., Wang R., Li N., Shen X., Zhou X., Lu C., Zheng X., Hu Z. (2009). NaCl-induced alternations of cellular and tissue ion fluxes in roots of salt-resistant and salt-sensitive poplar. Plant Physiol.

[27] Sun J., Dai S., Wang R., Chen S., Li N., Zhou X., Lu C., Shen X., Zheng X., Hu Z. (2009). Calcium mediates root K^+^/Na^+^ homeostasis in poplar species differing in salt tolerance. Tree Physiol.

[28] Sun J., Li L., Liu M., Wang M., Ding M., Deng S., Lu C., Zhou X., Shen X., Zheng X. (2010). Hydrogen peroxide and nitric oxide mediate K^+^/Na^+^ homeostasis and antioxidant defense in NaCl-stressed callus cells of two contrasting poplars. Plant Cell Tissue Organ Cult.

[29] Sun J., Wang M., Ding M., Deng S., Liu M., Lu C., Zhou X., Shen X., Zheng X., Zhang Z. (2010). H_2_O_2_ and cytosolic Ca^2+^ signals triggered by the PM H^+^-coupled transport system mediate K^+^/Na^+^ homeostasis in NaCl-stressed *Populus euphratica* cells. Plant Cell Environ.

[30] Shabala S., Cuin T.A. (2008). Potassium transport and plant salt tolerance. Physiol. Plant.

[31] Leplé J.C., Brasiliero A., Michel M.F., Delmotte F., Jouanin L. (1992). Transgenic poplars: Expression of chimeric genes using four different constructs. Plant Cell Rep.

[32] Kriz W., Schnermann J., Höhling H.J., Von Rosenstiel A.P., Hall T.A., Wirz H., Spinelli F. (1972). Electron probe microanalysis of electrolytes in kidney cells. Recent Advances in Renal Physiology.

